# Effects of post‐ovulatory aging on centromeric cohesin protection in murine MII oocytes

**DOI:** 10.1002/rmb2.12433

**Published:** 2021-12-13

**Authors:** Gaku Shimoi, Rico Wakabayashi, Ryu Ishikawa, Yuichi Kameyama

**Affiliations:** ^1^ Faculty of Bioindustry Tokyo University of Agriculture Abashiri Japan; ^2^ Graduate School of Bioindustry Tokyo University of Agriculture Abashiri Japan

**Keywords:** AURORA B, post‐ovulatory oocyte aging, PP2A, REC8, SGO2

## Abstract

**Purpose:**

Post‐ovulatory aging causes a high frequency of aneuploidy during meiosis II in mouse oocytes, irrespective of maternal age. In this study, we evaluated the effects of post‐ovulatory oocyte aging on the protection of chromosomal cohesion involved in aneuploidy and verified the relationship between PP2A or SGO2 expression and the phosphorylation level of REC8 in oocytes.

**Methods:**

Murine ovulated oocytes were incubated for 6 or 12 h *in vitro* after collection and denoted as the aged group. The oocytes examined immediately after collection were used as the control group. Immunofluorescent staining was used to detect the localization of PP2A, SGO2, BUB1, AURORA B, and MAD2 in the chromosomal centromere. Immunoblotting was used to quantify the expression of proteins describe above and REC8 in the oocytes.

**Results:**

PP2A expression involved in the de‐phosphorylation of REC8 decreased over time in oocytes, suggesting a deficiency in PP2A in centromeres. This indicated an increase in the level of phosphorylated REC8, which destabilizes centromeric cohesion in oocytes. In contrast, SGO2 expression was significantly high in aged oocytes.

**Conclusions:**

The findings show that post‐ovulatory aging destabilizes the centromeric cohesin protection in oocytes and can cause aneuploidy, which is often observed in aged oocytes during meiosis II.

## Graphical abstract

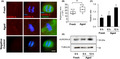

## 1 INTRODUCTION

It is well known that mammalian oocyte quality degrades over time after ovulation *in vitro*.^1^ To gain insight into oocyte aging, the aging process needs to be considered on two different axes of time. One is maternal aging, which involves an inter‐annual change in oocytes that advances with age. This is also described as pre‐ovulatory aging, which progresses in the ovary. The other is post‐ovulatory aging, the temporal alterations in oocytes that progress in the oviduct or can be induced *in vitro*. Most studies that investigated oocyte aging have focused on the effect of maternal age, that is, pre‐ovulatory aging. However, as the *in vitro* manipulation time for ovulated oocytes used in reproductive medicine and ova research lengthens, post‐ovulatory oocyte aging would become a problem that must be overcome. For example, rescue ICSI, which is often carried out in reproductive medicine, is a treatment program in which ICSI is performed on oocytes judged to be unfertilized after IVF to obtain fertilized oocytes. We believe that target oocytes used for rescue ICSI are already aged by the time of re‐insemination and are therefore essentially different from fresh unfertilized oocytes.

Chromosomal aneuploidy is a well‐known problem caused by oocyte aging.^1^, ^2^, ^3^ Chromosomal aneuploidy in oocytes, which is a major cause of poor developmental competence and loss of pregnancy,^1^, ^3^, ^4^, ^5^, ^6^, ^7^ results from chromosomal segregation errors during meiosis.^8^, ^9^ It is known that chromosomal segregation errors such as early segregation and nondisjunction occur during meiosis I in oocytes aged in ovaries with maternal aging.^10^, ^11^ Our previous study showed that post‐ovulatory aging also produces a high frequency of aneuploidy during meiosis II, irrespective of maternal age.^12^ The spindle assembly checkpoint (SAC), which functions during cell division, is one of the checkpoint mechanisms that function during the cell cycle. SAC is a monitoring system that equally distributes chromosomes by correctly attaching spindle microtubules to the chromosome kinetochore. We have reported that oocyte aging *in vitro* leads to the destabilization of SAC signaling and causes segregation errors in sister chromatids following the metaphase II (MII).^12^ Mammalian oocytes arrest meiosis in the MII stage before ovulation. In ovulated oocytes, it is necessary to align chromosomes on the equatorial plane and maintain adhesion between sister chromatids until meiosis is resumed by fertilization. The cohesin complex, which has a ring‐shaped protein structure, acts as an adhesion factor that maintains sister chromatid connections.^12^, ^13^, ^14^ In meiosis I, cohesin between sister chromatids in the arms of the two homologous chromosomes that form the divalent chromosome is resolved by degradative enzymes, whereas cohesin between sister chromatids in the centromere is maintained.^13^, ^14^ This mechanism ensures that sister chromatid pairs are correctly recognized and distributed to both poles during meiosis II. In MII oocytes, cohesin complexes that localize to the chromosomal centromere provide adhesion between sister chromatids. REC8, which contains a cleavage recognition site for separase, is one of the protein subunits of the meiotic cohesion complex. Degradation of REC8 by activated separase following the release of the SAC signal results in the dissociation of cohesin from the chromosomal centromere. We have shown that time‐dependent deterioration of REC8 occurs in murine oocytes aged *in vitro* after ovulation, despite before activation.^12^ This suggests that *in vitro* aging after ovulation weakens cohesion between sister kinetochores and causes a high frequency of aneuploidy after MII in oocytes.

Shugoshin protein was identified as a factor that protects cohesin REC8 on the centromere from separase so that sister chromatids do not segregate until the anaphase during meiosis.^15^ Two paralogs of Shugoshin, Sgo1 and Sgo2, have been identified in fission yeast.^16^ Sgo1, which is specifically expressed during meiosis I, localizes to the centromere and prevents sister chromatids from dissociation until meiosis I.^16^ In contrast, Sgo2, which is constantly expressed on the centromere during cell division (M‐phase), contributes to the accurate distribution of sister chromatids.^17^ It has been reported that Shugoshin regulates the phosphorylation level of the target protein during the M‐phase by interacting with protein phosphatase 2A (PP2A), which is a dephosphorylate enzyme.^18^ Cohesin protection is accomplished by the de‐phosphorylation of REC8, which is enabled by the recruitment of PP2A via Shugoshin to the chromosomal centromere.^15^, ^18^, ^19^ Shugoshin has been widely conserved from yeast to humans in eukaryotes, and an SGO‐like protein has been found in various eukaryotes^18^; this has been defined as the Shugoshin family protein. In humans and mice, two SGO‐like proteins, SGO1 and SGO2, have been found. Although the role of Shugoshin during meiosis in mammals seems to be similar to that in fission yeast, the behavior of Shugoshin is not fully understood. Functional analysis of Shugoshin using murine oocytes has indicated that SGO1 and SGO2 are expressed during both meiosis I and II, in contrast to the process in fission yeast.^20^ In addition, SGO2 is more highly expressed than SGO1 during the meiosis process, and it has been clarified that SGO2 is essential for the protection of centromeric cohesion.^15^, ^20^ Therefore, SGO2 and PP2A may be involved in the reduced expression of cohesin REC8 in MII oocytes, as found in previous studies.

The purpose of this study was to examine the effect of post‐ovulatory aging on the expression and localization of SGO2 and PP2A in murine oocytes, and to verify its relationship with the phosphorylation level of cohesin REC8.

## 2 MATERIALS AND METHODS

### 2.1 Animals

B6D2F1 male (10–15 weeks old) and female (8–10 weeks old) mice were used for the experiments described below. The mice were maintained under controlled conditions of light (14 h light from 6:00 to 20:00; 10 h dark) and temperature (24 ± 2°C). All the procedures involving animal experiments were conducted according to the guidelines approved by the Animal Research Committee of the Tokyo University of Agriculture.

### 2.2 Oocyte collection and in vitro aging

To obtain MII oocytes, female mice were induced to super‐ovulate by consecutive injection of 5 IU pregnant mare serum gonadotropin (ASKA Animal Health Co., Ltd.) and 5 IU human chorionic gonadotropin (hCG; ASKA Animal Health) at 48‐h intervals. The super‐ovulated mice were euthanized 15 h after the hCG injection, and their oviductal ampullae were broken to release the cumulus‐oocyte complexes (COCs) into TYH medium under paraffin liquid (Nacalai Tesque, Inc.). The collected COCs were incubated for a certain time at 37.5 °C under a humidified atmosphere with 5% CO_2_ and used as *in vitro* aged oocytes. Two experimental groups were designated in this study. One was an aged group, comprising oocytes incubated for 6 or 12 h *in vitro* after oocyte collection. In our previous study, it has already reported that aging time over 6 h *in vitro* increases numerical chromosome aberrations in oocytes during meiosis II. Especially, 40% of aged oocyte for 12 h *in vitro* has caused chromosome aneuploidy.^12^ The other was a fresh group, which functioned as the control, comprising oocytes that were immediately examined after collection.

### 2.3 Chromosome analysis

To eliminate the male genome factor, chromosome analysis of oocytes was performed after activating treatment by SrCl2 without fertilization. After the activation treatment, oocytes were cultured in mKSOM for 20 h. Six hours before termination of the culture, the activated oocytes were treated with mKSOM supplemented with 0.1 µg/ml demecolcine (Wako Pure Chemical Industries, Ltd.). Chromosome spreads were prepared according to the method reported by Yoshizawa et al.^21^ with slight modification. The slides were stained for 15 min with 4% Giemsa solution (Merck Millipore). Stained chromosome spreads were observed with an optical microscope, and the number of chromosomes was counted. Chromosome spreads with more or less than 40 chromosomes, or showing polyploidy, were considered as numerical chromosome aberrations (NCAs).

### 2.4 Immunofluorescent staining

Localization analysis of PP2A, SGO2, BUB1, AURORA B, and MDA2 protein in MII oocytes was performed using immunofluorescent staining. Ten oocytes were stained for each experimental group, and quantified fluorescence intensity. Immunofluorescent staining was performed once for each protein. The zona pellucida was removed from the oocytes with washes in Dulbecco's phosphate‐buffered saline (D‐PBS) containing 0.5% pronase (Merck Millipore) for 5 min at 37 °C, and fixed with 4% paraformaldehyde (Wako) in D‐PBS for 30 min at room temperature. After fixation, the oocytes were treated with 1% Triton X‐100 (Wako) in D‐PBS for 15 min at room temperature and washed thrice with D‐PBS. The permeabilized oocytes were blocked in 3% bovine serum albumin (Sigma Chemical Co.) for 30 min at room temperature. After washing thrice with D‐PBS, the oocytes were incubated with mouse anti‐PP2A‐Aα/β antibody (1:400 dilution, sc‐13600; Santa Cruz Biotechnology, Inc.), rabbit anti‐SGO2 antibody (1:400 dilution, MBS858924; My BioSource, Inc.), mouse anti‐BUB1 antibody (1:100 dilution, sc‐365685; Santa Cruz), mouse anti‐ARK‐2 antibody (1:100 dilution, sc‐393357; Santa Cruz), or goat anti‐MAD2 antibody (1:100 dilution, sc‐6329; Santa Cruz) as the primary antibody overnight at 4°C and then again washed thrice with D‐PBS. This was followed by incubation with Alexa Fluor^®^ 594‐conjugated anti‐mouse IgG secondary antibody (1:800 dilution, #8890; Cell Signaling Technology, Inc.), Alexa Fluor^®^ 488‐conjugated anti‐rabbit IgG (1:800 dilution, 111–545–003; Jackson ImmunoResearch Laboratories, Inc.), or Alexa Fluor^®^ 594‐conjugated anti‐goat IgG secondary antibody (1:200 dilution, 705–585–003; Jackson ImmunoResearch Laboratories) for 60 min at room temperature. Oocyte chromosomes were stained with 1 μg/ml Hoechst33258 (Sigma) for 30 min at 4°C. Following washing, the oocytes were mounted on glass slides, covered with glass cover slips, and observed under 200–400× magnification using a fluorescence microscope system, BX51 and DP71 (Olympus Co.). ImageJ was used to quantify the signal intensity of the detected target protein. The captured RGB images containing the signal of target protein were converted to grayscale, and the sum of the gray values of all the pixels in the region indicated by the signal was taken as the brightness value.

### 2.5 Immunoblotting

The expression of REC8, PP2A, SGO2, BUB1, AURORA B, and MAD2 proteins was detected in MII oocytes using immunoblotting. In this study, parthenogenetic oocytes were excluded from the analyzed samples of oocytes. 120 oocytes/tube were sampled for each experimental group, and immunoblotting was repeated 5 times for each protein. Oocytes were lysed in RIPA buffer (Wako) supplemented with a protease inhibitor cocktail (Thermo Fisher Scientific K.K.) and incubated on ice for 10 min. After centrifugation at 12,000 × *g* for 5 min at 4°C, the oocyte lysates were frozen at −80°C until use. The oocyte lysates were diluted with an equal volume of 2× Laemmli sample buffer (Bio‐Rad) containing 5% 2‐mercaptoethanol (Wako) and heated to 100°C for 5 min. Proteins were separated using sodium dodecyl sulfate polyacrylamide gel electrophoresis (SDS‐PAGE) with a stacking gel containing 4% acrylamide (Wako) and a separating gel containing 7.5–10% acrylamide run for 50 min at 200 V. Proteins were then electrophoretically transferred onto polyvinylidene difluoride (PVDF) membranes (GE Healthcare) for 30 min at 15 V. Each membrane was blocked with PVDF blocking reagent (TOYOBO Co., Ltd.) for 1 h at room temperature, followed by incubation overnight at 4 °C with rabbit anti‐p‐REC8 antibody (1:1,000 dilution, LS‐C47114; LifeSpan Biosciences, Inc.), mouse anti‐PP2A‐Aα/β antibody (1:1,000 dilution; Santa Cruz), rabbit anti‐SGO2 antibody (1:1,000 dilution; My BioSource), mouse anti‐BUB1 antibody (1:500 dilution; Santa Cruz), mouse anti‐ARK‐2 antibody (1:500 dilution; Santa Cruz), or goat anti‐MAD2 antibody (1:500 dilution; Santa Cruz) as the primary antibody. The membranes were then washed thrice with PBS containing 1% Tween 20 (PBS‐T) and incubated with a horseradish peroxidase (HRP)‐conjugated anti‐rabbit IgG (1:2,000 dilution, NA934; GE Healthcare), anti‐mouse IgG (1:2,000 dilution, NA931; GE Healthcare), or anti‐goat IgG (1:1,000 dilution, sc‐2304; Santa Cruz) secondary antibody for 1 h at room temperature. The membranes were washed thrice with PBS‐T, and proteins were detected using an ECL Prime western blotting detection kit (GE Healthcare). After the detection of target proteins, the membranes were washed twice and re‐blocked. As an internal control, the expression of α‐tubulin or β‐actin was detected, as described above, using mouse anti‐α‐tubulin antibody (1:1,000 dilution, 017–25031; Wako) or mouse anti‐β‐actin antibody (1:1,000 dilution, 013–24553; Wako) as the primary antibody and HRP‐conjugated anti‐mouse IgG as the secondary antibody (1:2,000 dilution; GE Healthcare). ImageJ was used to quantify the intensity of the protein bands of interest, and band intensities were normalized to an internal control.

### 2.6 Statistical analysis

The Chi‐square test was used to determine the statistical significance of the percentages of NCAs. The Student's *t*‐test was used to compare the average brightness values of fluorescent signal between aged and fresh group. The one‐way ANOVA, followed by Tukey‐Kramer test of multiple comparisons, was used to compare the average expression levels of proteins among groups. Statistical significance was set at *p* < 0.05.

## 3 RESULTS

### 3.1 Numerical chromosome aberration in oocytes

**FIGURE 1:**
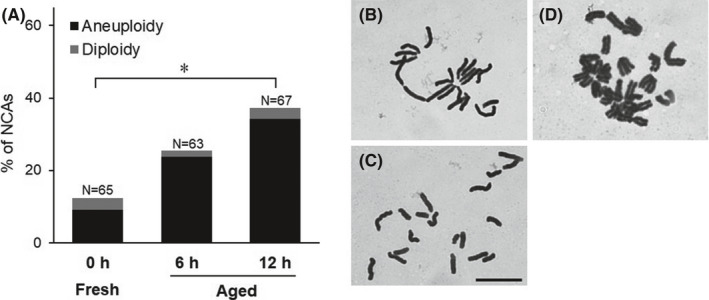
Comparison of the frequency of NCAs between aged and fresh groups. A, Frequency of NCAs in mouse oocytes. B, Normal chromosome spread in mouse oocyte (*n* = 20). C, Chromosome spread which shows aneuploidy in mouse oocyte (*n* = 19). D, Chromosome spread which shows diploidy in mouse oocyte (*n* = 40). Scale bar = 10 µm. * *p *< 0.05

The rates of NCAs in the post‐ovulatory aged oocytes completing second meiosis are shown in Figure 1. NCAs were detected in 25.4% (16/63) and 37.3% (25/67) of all observed chromosome spreads in the 6‐ and 12‐h aged groups, respectively, and a number of NCAs in the 12‐h aged group were significantly higher than that in the fresh group (12.3%, 8/65) (*p* < 0.05). Most of the NCAs observed in oocytes indicated aneuploidy (23.8, 34.3% in 6‐ and 12‐h aged groups respectively, and 9.2% in the fresh group). There was no difference of brightness values of SGO2 signal between the aged group and the fresh group (Figure 4B).

### 3.2 Expression of p‐REC8 and t‐REC8

**FIGURE 2:**
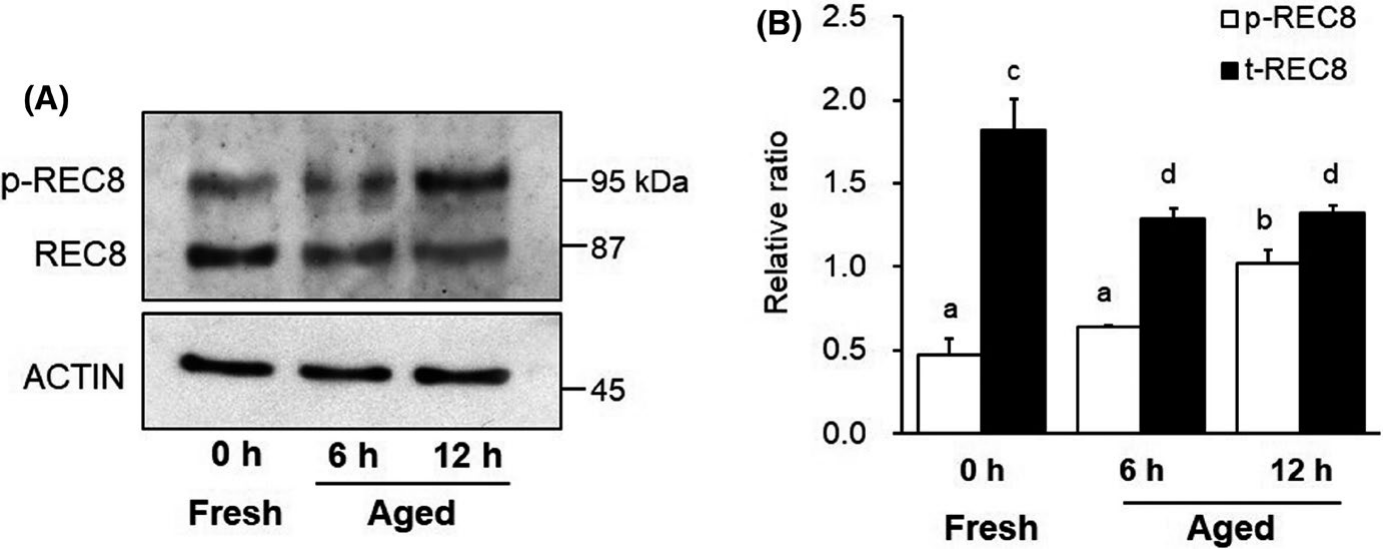
Comparison of expression levels of phosphorylated REC8 (p‐REC8) and total REC8 (t‐REC8) in mouse oocytes. A, Western blotting for p‐REC8 and t‐REC8 proteins in MII oocyte. β‐actin was used as a loading control. B, Relative expression of p‐REC8 or t‐REC8 to β‐actin. Bars with different superscripts indicate significant differences (*p* < 0.05)

The expression levels of phosphorylated REC8 (p‐REC8) and total REC8 (t‐REC8) in MII oocytes are shown as ratios, relative to β‐actin expression, in Figure 2A,B. The expression levels of p‐REC8 were 0.64 ± 0.01 and 1.03 ± 0.08 in the 6‐ and 12‐h aged groups, respectively. The p‐REC8 level of MII oocytes increased over time *in vitro*, with a significant difference between the 12‐h aged group and the fresh group (0.48 ± 0.10, *p* < 0.05). The expression levels of t‐REC8, including un‐phosphorylated REC8, were 1.29 ± 0.06 and 1.33 ± 0.04 in the 6‐ and 12‐h aged groups, respectively. There were significant differences between the aged groups and the fresh group (1.82 ± 0.18, *p* < 0.05).

### 3.3 Expression of PP2A

**FIGURE 3:**
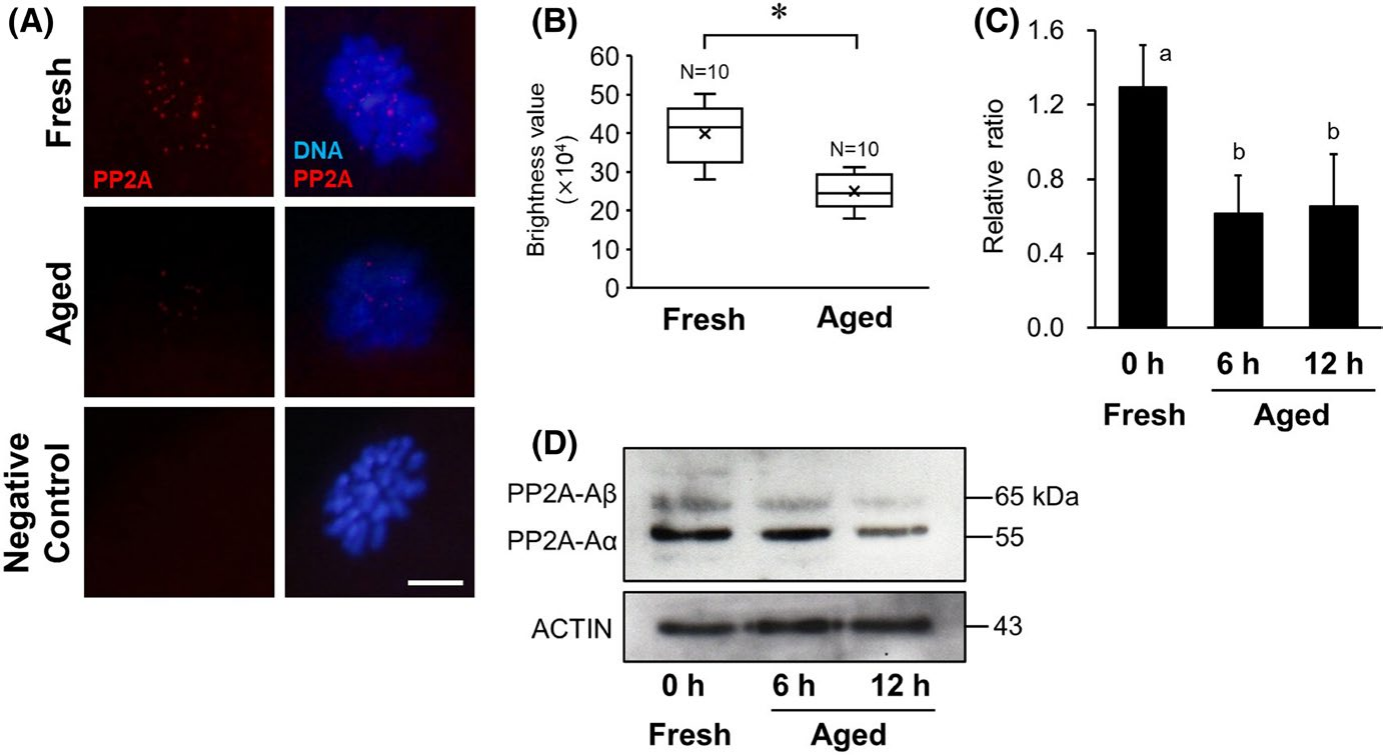
Comparison of PP2A expression in mouse oocytes. A, Localization of PP2A in mouse oocytes. Immunofluorescent staining of PP2A (red) in the 12‐h aged and fresh oocytes. DNA (blue) was stained with Hoechst 33258. D‐PBS was used as the negative control instead of the primary antibody. Scale bar = 10 μm. B, Comparison of brightness values of PP2A signal in the 12‐h aged and fresh oocytes. *p < 0.05. C, Relative expression of PP2A to β‐actin. Bars with different superscripts indicate significant differences (*p* < 0.05). D, Western blotting for PP2A‐Aα and Aβ subunit proteins in MII oocyte. β‐actin was used as a loading control

The immunofluorescent staining results of PP2A‐A in MII oocytes are shown in Figure 3A. PP2A localization was clearly detected in chromosome kinetochores in the fresh group. Although PP2A signals were also detected in chromosome kinetochores in the aged group, the brightness values of PP2A signal were significantly lower than that in the fresh group (*p* < 0.05, Figure 3B). The expression levels of PP2A in MII oocytes are shown in Figure 3C,D. In this study, we assessed the expression of PP2A‐A, which is a PP2A structural A subunit containing two isoforms (Aα and Aβ), as an indicator of PP2A complex expression. The expression levels of PP2A‐A are shown as ratios, relative to β‐actin expression, and were 0.62 ± 0.20 and 0.66 ± 0.28 in the 6‐ and 12‐h aged groups, respectively. There were significant differences between the aged groups and the fresh group (1.29 ± 0.22, *p* < 0.05, Figure 3C).

### 3.4 Expression of SGO2

**FIGURE 4:**
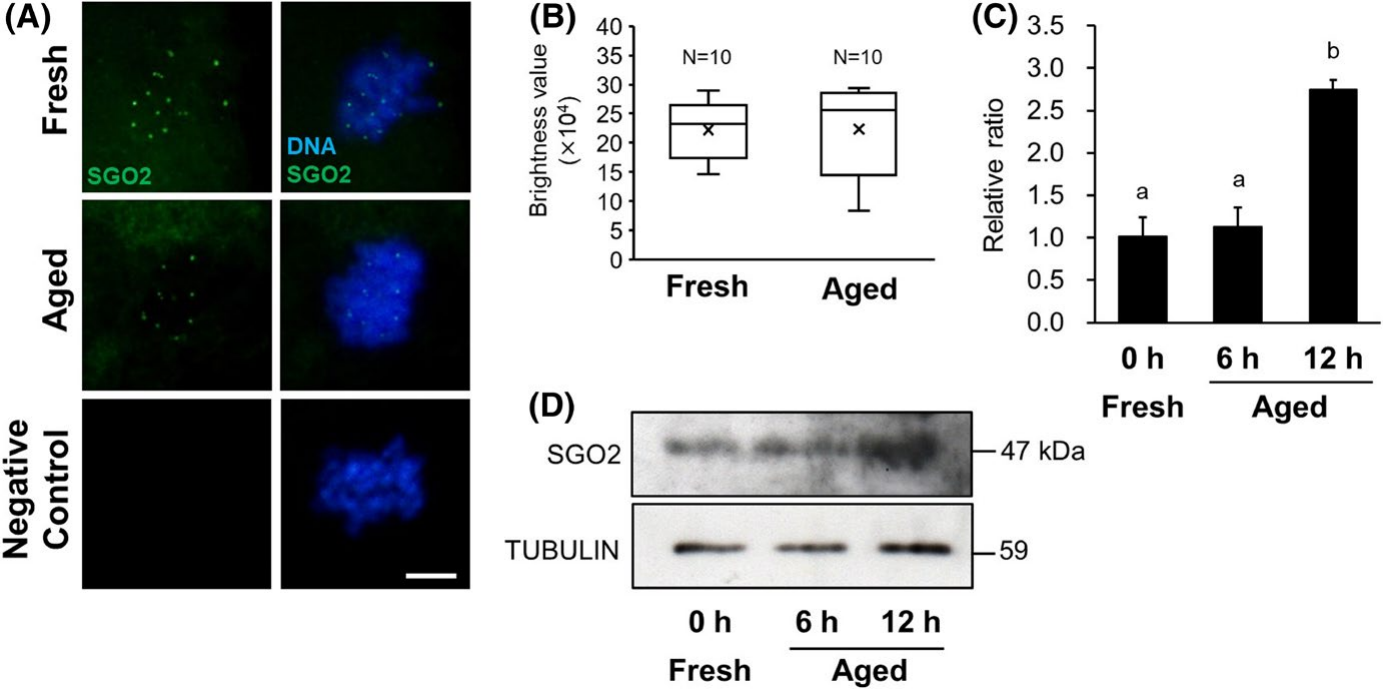
Comparison of SGO2 expression in mouse oocytes. A, Localization of SGO2 in mouse oocytes. Immunofluorescent staining of SGO2 (green) in the 12‐h aged and fresh oocytes. DNA (blue) was stained with Hoechst 33258. D‐PBS was used as the negative control instead of the primary antibody. Scale bar = 10 μm. B, Comparison of brightness values of SGO2 signal in the 12‐h aged and fresh oocytes. C, Relative expression of SGO2 to α‐tubulin. Bars with different superscripts indicate significant differences (*p* < 0.05). D, Western blotting for SGO2 protein in MII oocyte. α‐tubulin was used as a loading control

Immunofluorescent staining results for SGO2 in MII oocytes are shown in Figure 4A. SGO2 localization was clearly detected in chromosome kinetochores, and there was no difference of brightness values of SGO2 signal between the aged group and the fresh group (Figure 4B). The expression levels of SGO2 in MII oocytes are shown in Figure 4C,D. In post‐ovulatory aged oocytes, the expression levels of SGO2 are shown as ratios, relative to α‐tubulin expression, and were 1.14 ± 0.22 and 2.75 ± 0.11 in the 6‐ and 12‐h aged groups, respectively. There was a significant difference between the 12‐h aged group and the fresh group (1.02 ± 0.22, *p* < 0.05, Figure 4C).

### 3.5 Expression of BUB1

**FIGURE 5:**
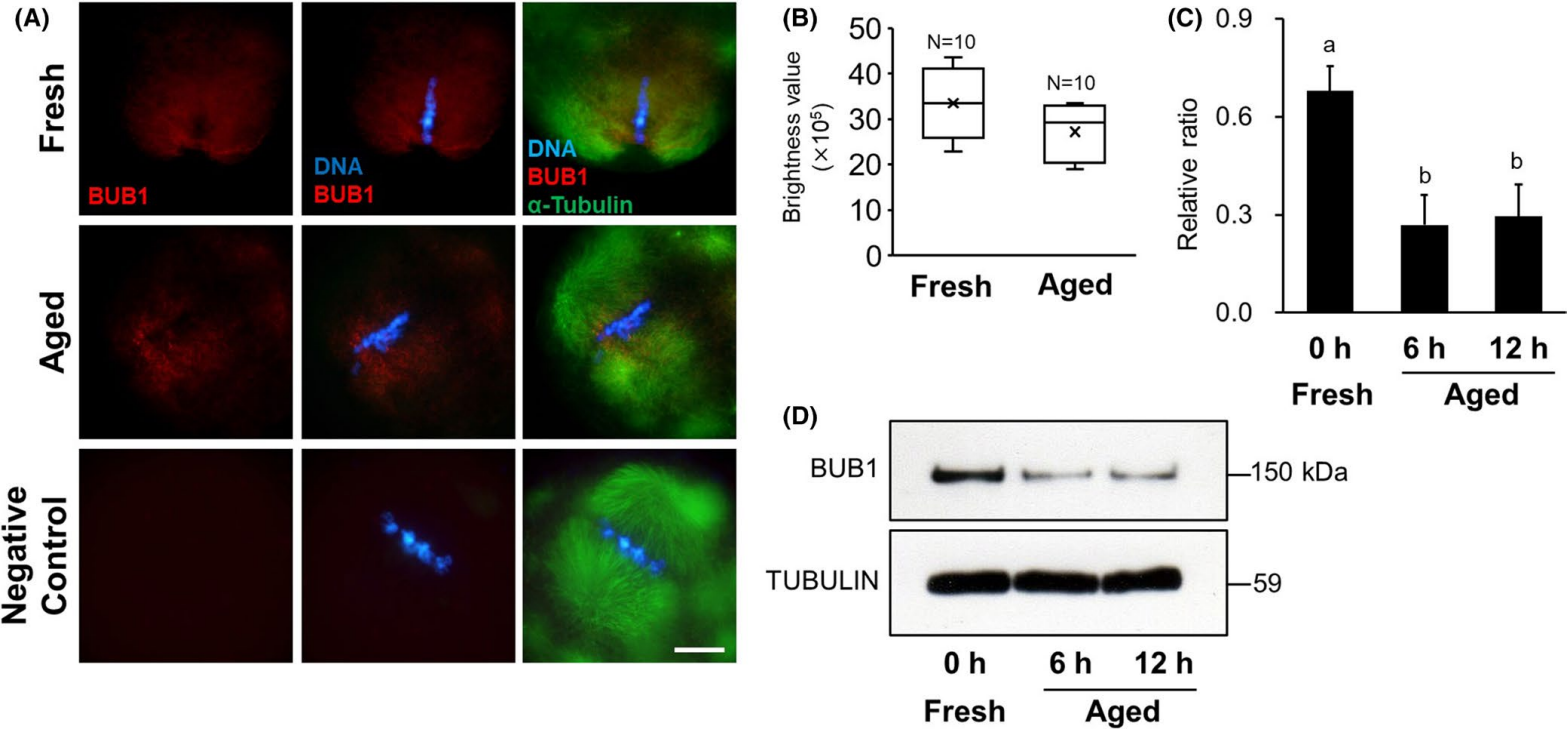
Comparison of BUB1 expression in mouse oocytes. A, Localization of BUB1 in mouse oocytes. Immunofluorescent staining of BUB1 (red) and α‐tubulin (green) in the 12‐h aged and fresh oocytes. DNA (blue) was stained with Hoechst 33258. D‐PBS was used as the negative control instead of the primary antibody. Scale bar = 10 μm. B, Comparison of brightness values of BUB1 signal in the 12‐h aged and fresh oocytes. C, Relative expression of BUB1 to α‐tubulin. Bars with different superscripts indicate significant differences (*p* < 0.05). D, Western blotting for BUB1 protein in MII oocyte. α‐tubulin was used as a loading control

Immunofluorescent staining results for BUB1 in MII oocytes are shown in Figure 5A. The brightness values of BUB1 signal in the aged group tend to be lower than that in the fresh group, but there was no difference of brightness values of BUB1 signal between the aged group and the fresh group (Figure 5B). The expression levels of BUB1 in MII oocytes are shown in Figure 5C,D. In post‐ovulatory aged oocytes, the expression levels of BUB1 are shown as ratios, relative to α‐tubulin expression, and were 0.27 ± 0.09 and 0.30 ± 0.10 in the 6‐ and 12‐h aged groups, respectively. There was a significant difference between the aged group and the fresh group (0.68 ± 0.07, *p* < 0.05, Figure 5C).

### 3.6 Expression of AURORA B

**FIGURE 6:**
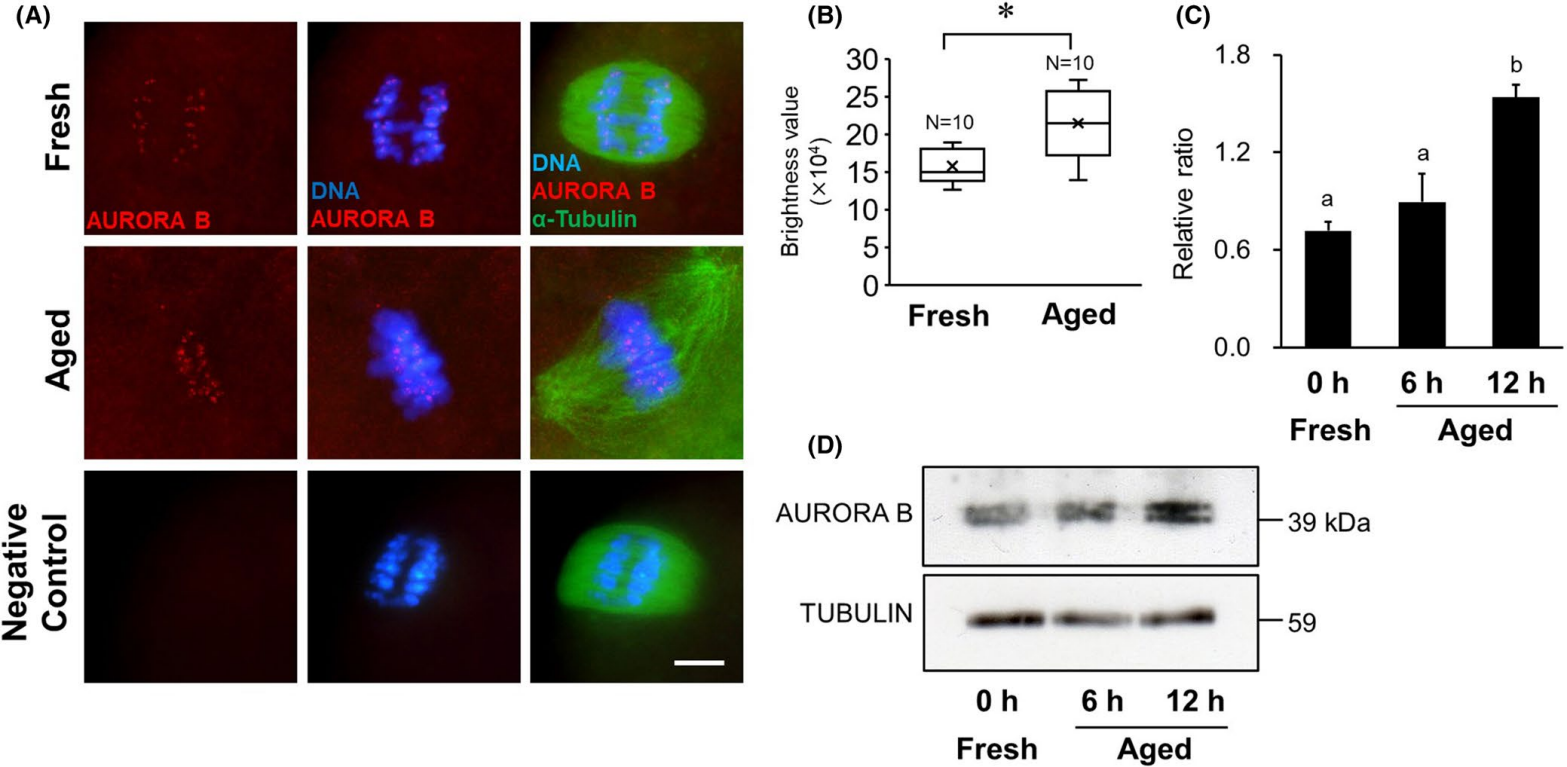
Comparison of AURORA B expression in mouse oocytes. A, Localization of AURORA B in mouse oocytes. Immunofluorescent staining of AURORA B (red) and α‐tubulin (green) in the 12‐h aged and fresh oocytes. DNA (blue) was stained with Hoechst 33258. D‐PBS was used as the negative control instead of the primary antibody. Scale bar = 10 μm. B, Comparison of brightness values of AURORA B signal in the 12‐h aged and fresh oocytes. * *p* < 0.05. C, Relative expression of AURORA B to α‐tubulin. Bars with different superscripts indicate significant differences (*p* < 0.05). D, Western blotting for AURORA B protein in MII oocyte. α‐tubulin was used as a loading control

Immunofluorescent staining results for AURORA B in MII oocytes are shown in Figure 6A. Although AURORA B localization was clearly detected in chromosome kinetochores in both aged and fresh group, the brightness values of AURORA B signal in the 12‐h aged group were significantly higher than that in the fresh group (*p* < 0.05, Figure 6B). The expression levels of AURORA B in MII oocytes are shown in Figure 6C,D. In post‐ovulatory aged oocytes, the expression levels of AURORA B are shown as ratios, relative to α‐tubulin expression, and were 0.90 ± 0.17 and 1.54 ± 0.08 in the 6‐ and 12‐h aged groups, respectively. There was a significant difference between the 12‐h aged group and the fresh group (0.72 ± 0.05, *p* < 0.05, Figure 6C).

### 3.7 Expression of MAD2

**FIGURE 7:**
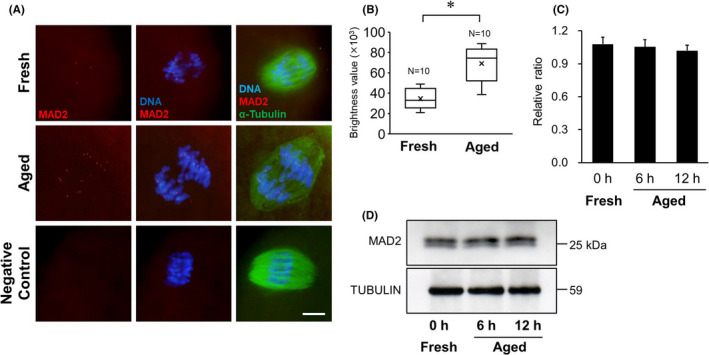
Comparison of MAD2 expression in mouse oocytes. A, Localization of MAD2 in mouse oocytes. Immunofluorescent staining of MAD2 (red) and α‐tubulin (green) in the 12‐h aged and fresh oocytes. DNA (blue) was stained with Hoechst 33258. D‐PBS was used as the negative control instead of the primary antibody. Scale bar = 10 μm. B, Comparison of brightness values of MAD2 signal in the 12‐h aged and fresh oocytes. * *p* < 0.05. C, Relative expression of MAD2 to α‐tubulin. D, Western blotting for MAD2 protein in MII oocyte. α‐tubulin was used as a loading control

The immunofluorescent staining results of MAD2 in MII oocytes are shown in Figure 7A. Although MAD2 signals were clearly detected in chromosome kinetochores in both aged and fresh group, the brightness values of MAD2 signal in the aged group were significantly higher than that in the fresh group (*p* < 0.05, Figure 7B). The expression levels of MAD2 in MII oocytes are shown in Figure 7C,D. In post‐ovulatory aged oocytes, the expression levels of MAD2 are shown as ratios, relative to α‐tubulin expression, and were 1.05 ± 0.07 and 1.02 ± 0.05 in the 6‐ and 12‐h aged groups, respectively. There was no difference of expression levels of MAD2 signal between the aged group and the fresh group (1.08 ± 0.06, Figure 7C).

## 4 DISCUSSION

In this study, three facts about the effect of post‐ovulatory oocyte aging on the protective mechanism of cohesin in MII were revealed. Firstly, the phosphorylation level of REC8 protein increases over time after ovulation in MII oocytes. Secondly, the expression of PP2A, which is involved in the de‐phosphorylation of REC8, begins to decrease in oocytes 6 h after oocyte collection, suggesting a deficiency in PP2A in centromeres. Finally, SGO2, which recruits PP2A to the chromosome centromere, is overexpressed and might act as a versatile centromere adapter to recruit another protein to centromere in aged oocytes. These facts suggest a destabilization of the cohesin‐protective mechanism in oocytes.

In present study, it was showed that the NCA frequencies in oocyte, which had completed second meiosis, increase with advancing aging time. Most of the NCAs observed from the aged oocyte exhibited aneuploidy as in previous reports. The major factor causing chromosomal aneuploidy in oocytes is abnormality of chromosome separation during meiosis. Cohesin complexes act as adhesion molecules that connect sister chromatids during chromosome segregation regulated by the SAC. REC8, which is a meiosis‐specific subunit of cohesin, has a separase recognition site that is specifically cleaved by separase.^22^ Therefore, the expression of cohesin REC8 is essential to retain proper sister chromatid connections until the terminal phase of chromosome segregation. It is known that *Rec8*‐deficient mice show a loss in synapsis in homologous chromosomes and a poor cohesion between sister chromatids.^23^ Even in wild‐type mice, it has been shown that REC8 levels gradually decrease on the chromosome as mice age.^22^, ^23^, ^24^, ^25^ In old mice, low levels of REC8 expression and weakened cohesion of sister chromatids have been recognized in oocytes with poor connections between sister kinetochores.^24^ Decreases in the expression of cohesin REC8 in oocytes are not solely due to advances in maternal age. Mammalian oocytes are arrested in MII prior to ovulation and maintain the MII stage until fertilization is complete. In our previous study, we showed that the expression level of REC8 in young murine oocytes decreased over time after ovulation until fertilization was completed.^12^ This result indicates that post‐ovulatory aging *in vitro* can reduce REC8 expression in MII oocytes in a time‐dependent manner, even at a young age, irrespective of maternal age. The new finding in the present study was that the expression level of p‐REC8 increased in a time‐dependent manner after ovulation, in contrast to that of t‐REC8. To maintain the function of cohesin as an adhesion factor between sister chromatids, REC8 must be de‐phosphorylated and protected from degrading enzymes.^15^ Therefore, *in vitro* aging after ovulation promotes the degradation of REC8 by separase following the phosphorylation of REC8 in MII oocytes. Indeed, in this study, we demonstrated that the expression level of t‐REC8 in oocytes aged for more than 6 h after collection from the oviduct was significantly lower than that in fresh oocytes immediately after collection, as in a previous study.

Two factors, PP2A and SGO2, are involved in the de‐phosphorylation of REC8, which is the protective mechanism of cohesin. PP2A is a major intracellular phosphatase of serine/threonine protein, which regulates extensive cell signaling, including cell cycle and apoptosis.^26^ PP2A localized in the chromosomal centromere is a factor that performs REC8 de‐phosphorylation. In this study, although PP2A was localized on the chromosomal centromere of MII oocytes in both the aged and control groups, the results of brightness analysis indicate that the PP2A signals on the centromere were weaker in aged oocytes than in control oocytes. The accumulation of PP2A on the centromere may be insufficient in *in vitro* aged oocytes. PP2A holoenzymes are composed of three subunits: a structural scaffold A subunit, a regulatory B subunit, and a catalytic C subunit, forming a heterotrimer complex.^15^, ^26^, ^27^ Each subunit of PP2A has several isoforms; therefore, various combinations of subunits play important roles in regulating the localization and specific activity of PP2A.^27^ Oocyte‐specific deletion of the PP2A‐Aα isoform leads to precocious sister chromatid separation.^28^ However, unlike the loss of PP2A‐Aα, defects caused by double knockout of PP2A‐Cα and PP2A‐Cβ do not induce premature loss of centromeric cohesion, but cause an MI arrest associated with abnormal spindles and misaligned chromosomes.^27^ Thus, depletion of the PP2A‐Aα subunit causes the loss of centromere cohesion,^18^, ^29^ but the depletion of other subunits has different consequences. Therefore, much consideration should be given to the subunit composition of the relevant PP2A complex. In this study, the expression levels of PP2A‐Aα and PP2A‐Aβ in MII oocytes were examined, and it was shown that *in vitro* aging for 6 h or more significantly reduced the expression levels of PP2A. This supports the consideration that the accumulation of PP2A in the chromosomal centromere of *in vitro* aged oocytes may be deficient. It also suggests that a decrease in aging time‐related expression of PP2A and the depletion of PP2A in the centromere leads to an increase in the phosphorylation level of REC8, as described above.

SGO2 is a Shugoshin protein required to protect centromere cohesin during MI and MII in oocytes and is responsible for recruiting PP2A to centromeres by binding to it.^18^, ^19^, ^30^ In this study, SGO2 localization was observed on the chromosomal centromere in aged oocytes, similar to that in control oocytes. Localization of SGO2 depends on BUB1, a kinetochore kinase, and H2A, which is phosphorylated to BUB1.^31^ In fission yeast, it has been shown that inhibition of BUB1 reduces cohesin REC8 levels, and the existence of both BUB1 and SGO2 protects cohesin REC8.^32^ These results suggest that BUB1 is important for the activation of the protective mechanism of REC8 by SGO2. It is known that BUB1 expression gradually decreases with advancing maternal age in human oocytes.^32^ In this study, it was showed that BUB1 expression in *in vitro* aged oocytes also significantly decreased with advancing aging time. Despite the decline in PP2A and BUB1 recruitment to chromosome centromeres, the expression level of SGO2 was more than two times higher in the 12 h aged oocytes *in vitro* than in the control oocytes, contrary to our expectation. Many reports on the association between oocyte aging and SGO2 expression have been discussed from the viewpoint of maternal aging, and it has been suggested that SGO2 expressed in oocytes is gradually depleted in an age‐dependent manner, increasing the vulnerability of cohesin protein toward degradation.^22^, ^33^ In mice, PP2A does not localize to centromeres when SGO2 in oocytes is suppressed using a siRNA technique or when *Sgo2* is disrupted by gene knockout, indicating that SGO2 is required for PP2A recruitment to the centromere.^15^, ^20^, ^34^ Conversely, it has been reported that forced overexpression of *Sgo* in oocytes extends the localization of SGO and PP2A to chromosomal arms and enhances the capacity of SGO‐PP2A to protect cohesion.^15^, ^30^ SGO2 overexpression in post‐ovulatory aged oocytes may be associated with a function different from the selective protection of centromere cohesion between sister chromatids. Recent studies using fission yeast have shown that Shugoshin is involved in the localization of the chromosomal passenger complex (CPC) to the centromere, which plays an important role in chromosome alignment and contributes to the equal distribution of chromosomes.^15^, ^31^, ^35^ To achieve a chromosomal alignment and an equal distribution, the sister kinetochores must be pulled by spindle microtubules extending from opposite poles,^20^, ^31^, ^36^ which requires an accurate regulation of phosphorylation in kinetochore proteins.^37^ Aurora B kinase, a component of the CPC, destabilizes unsuitable attachment between kinetochores and microtubules by phosphorylating kinetochore proteins that act as interfaces in the kinetochore‐microtubule connection.^17^, ^37^ Conversely, phosphatase PP2A stabilizes attachments between kinetochores and microtubules by antagonizing the action of Aurora B and counteracting phosphorylation in the kinetochores.^37^ Thus, the phosphorylation level in kinetochores is finely adjusted by the balance between the activities of both Aurora B and PP2A. In resolving and correcting any misconnections between kinetochores and microtubules, SGO2 acts as a versatile centromere adapter that recruits CPC and MAD2, in addition to PP2A, to the centromere.^18^, ^37^ The overexpression of SGO2 observed in *in vitro* aged oocytes may have been induced compensatory to resolve the frequent misconnections between kinetochores and microtubules. In our present study, the expression levels and the localization signals of AURORA B increased in the aged oocyte group, which aneuploidy occurred frequently. Furthermore, although there is no difference of the expression levels of MAD2, more signals indicating MAD2 localization were identified on the chromosomal centromere in aged oocyte. MAD2 plays a role in monitoring the connection between spindle fibers and kinetochores,^38^, ^39^, ^40^ does not usually appear when connections between spindle fibers and kinetochores occur normally. Because MAD2 exists in a free state, not only in the nucleus but also in the cytoplasm,^38^ data assessing increases or decreases in MAD2 expression do not address MAD2's role as a checkpoint signal. However, based on the combination of MAD2 localization data and expression levels, it is possible to discern a potential age‐related dysfunction in the checkpoint system through which MAD2 monitors the kinetochore‐microtubule connection. Comprehensive consideration from these results, it is suggested that unsuitable attachment between kinetochores and microtubules occurs frequently in *in vitro* aged oocytes. A decrease in PP2A expression in aged oocytes is apparent, an existence of AURORA B kinase, which is highly expressed to correct the misconnections spindle‐kinetochore, might cause a deficiency in PP2A antagonizing the action of Aurora B. Therefore, it is speculated that the overexpression of SGO2 was caused by active recruitment of CPC to the kinetochores and maintenance of kinetochore proteins at high phosphorylation levels. To assess the overexpression of SGO2 observed in *in vitro* aged oocytes, it is necessary to verify the interaction between the protective function of cohesin and the attachment mechanism of kinetochores‐microtubules.

In conclusion, it was shown that post‐ovulatory oocyte aging reduces the expression level of phosphatase PP2A, suggesting a deficiency in PP2A in centromeres. Furthermore, it was revealed that the decrease in PP2A expression over time leads to an increase in the phosphorylation level of cohesin REC8 in MII oocytes. These findings show that post‐ovulatory aging destabilizes the protective mechanism of cohesin in oocytes, causing aneuploidy, which is often observed in *in vitro* aged oocytes during MII.

## CONFLICT OF INTEREST

Gaku Shimoi, Rico Wakabayashi, Masako Suzuki, Kei Shimada, Ryu Ishikawa, and Yuichi Kameyama declare that they have no conflict of interest.

## HUMAN RIGHTS

This article does not contain any studies with human subjects performed by the any of the authors.

## ANIMAL STUDIES

All institutional and national guidelines for the care and use of laboratory animals were followed.

## ETHICS APPROVAL

The study design and proposed studies were approved by the Animal Research Committee of the Tokyo University of Agriculture. All the procedures involving animal experiments were conducted according to the guidelines stipulated by the Animal Research Committee of the Tokyo University of Agriculture.
